# Bambusuril analogs based on alternating glycoluril and xylylene units

**DOI:** 10.3762/bjoc.15.124

**Published:** 2019-06-11

**Authors:** Tomáš Lízal, Vladimír Šindelář

**Affiliations:** 1 Department of Chemistry and RECETOX, Masaryk University, Kamenice 5, 625 00 Brno, Czech Republic

**Keywords:** bambusurils, conformers, glycolurils, macrocycles, supramolecular chemistry

## Abstract

The glycoluril monomer is a popular building block in supramolecular chemistry as it is used for the synthesis of versatile host molecules which can interact with cationic, anionic or neutral guest molecules. Here we present the design and synthesis of a new hybrid macrocycle containing glycoluril and aromatic units. The reaction afforded a mixture of macrocyclic homologues from which a two-membered macrocycle was isolated as the main product. Two disastereomers of the macrocycle were separated and characterized by means of NMR spectroscopy and X-ray crystallography. Conformational changes of these diastereomers were investigated using DFT models and variable-temperature NMR.

## Introduction

Macrocycles consisting of urea building blocks play an important role in supramolecular chemistry [1]. Urea N–H motifs provide macrocycles with the ability to act as anion receptors due to the stabilizing effect of N–H···anion hydrogen bonding [2–4]. Furthermore, the urea groups can participate in intermolecular hydrogen bonding resulting in the formation of tubular [5–7] or gel-like [8] structures. Ureas lacking of N–H hydrogen atoms such as ethyleneureas, glycolurils, and biotin are also important building blocks of macrocyclic receptors such as cucurbiturils [9] and hemicucurbiturils [10].

Bambus[6]urils are a special case of hemicucurbiturils, which comprise six glycoluril units connected by six methylene bridges within their structure [11–12]. Due to their hydrophobic cavity with 12 methine hydrogen atoms available for hydrogen bonding, bambus[6]urils bind inorganic anions with high binding affinity and selectivity in both organic media and water [13–14]. Previously we used bambusurils to recognize and quantify anions in their complex mixtures at sub-micromolar concentrations by means of NMR [15]. However, sensing of anions by less expensive UV–vis spectroscopy was not possible due to the low absorption of bambusurils within the UV–vis region. Therefore, we decided to investigate the synthesis of bambusuril derivatives and analogs containing chromophores in their structure. Initially we tested post-macrocyclic functionalization resulting in bambusurils with chromophore groups on their portals. However, the changes in the absorption spectra of these receptors upon anion binding were negligible since the groups on the portals were too distant from the anion binding site in the center of macrocycle. A different approach for the preparation of UV–vis-active bambusurils is based on the incorporation of the absorbing moiety directly into the macrocyclic framework. In such receptors, anion binding would take place in proximity of the chromophoric part of the macrocycle securing proper functioning of the sensor. Following this line, we decided to investigate the synthesis of bambusuril analogs in which glycoluril and chromophoric units alternate. Here we describe the first results of our efforts.

## Results and Discussion

**Scheme 1:**
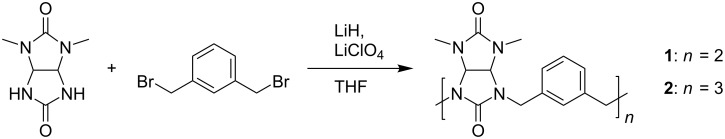
Reaction of 2,4-dimethylglycoluril and *m*-xylylene dibromide.

The preparation of the macrocycles was based on a one-pot reaction of 2,4-dimethylglycoluril and *m*-xylylene dibromide, a structurally simple example of a chromophoric monomer (Scheme 1). The reaction took place under basic conditions using LiH in dry THF [16]. The LiClO_4_ salt was used originally as a template in order to drive the reaction towards the three-membered macrocycle (*n* = 3). Later it was found that the salt does not have a significant effect on the distribution of macrocycle homologues probably due to the non-reversible nature of the reaction. However, the salt was used as it facilitated precipitation of the crude product after aqueous work-up. The MALDI analysis (Supporting Information File 1, Figure S1) indicated that the reaction yielded a mixture of two (*n* = 2) to five (*n* = 5)-membered macrocycles and acyclic oligomers.

**Figure 1:**
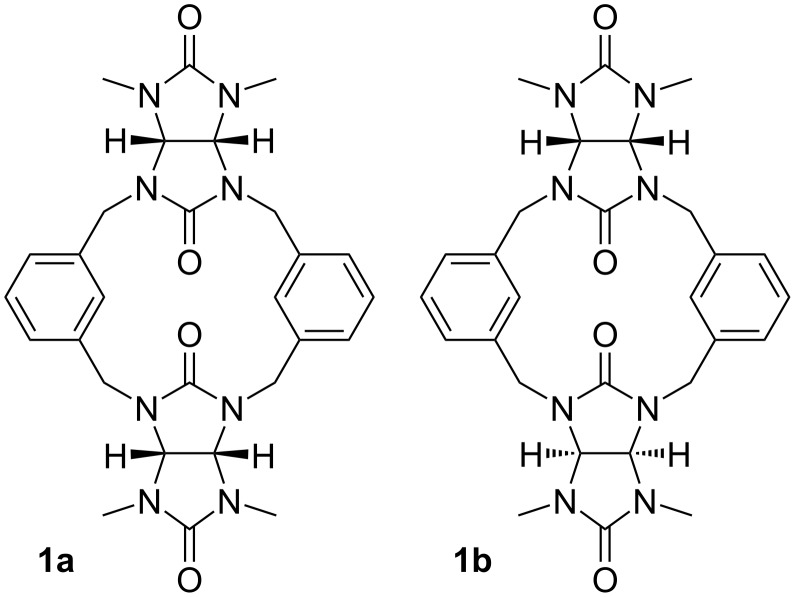
Molecular structures of **1a** and **1b**.

The crude mixture of macrocycles was separated by reversed-phase flash silica gel chromatography after normal phase silica and size-exclusion chromatography did not show satisfactory results. The separation was achieved using gradient elution with acetonitrile/water mixture. HPLC–MS analysis (Supporting Information File 1, Figure S2) revealed that the main product of the reaction is the two-membered macrocycle **1** (*n* = 2) followed by a small amount of the three-membered macrocycle **2** (*n* = 3). The chromatogram (Supporting Information File 1, Figure S2) showed two peaks with the same *m*/*z* value corresponding to **1** which indicated the presence of two diastereoisomeric forms **1a** and **1b** (Figure 1). The isomerism arises from the two possible orientations of glycoluril units within the macrocycle. The rigid structure of glycoluril features methine protons on its convex face which can either point to the same or the opposite direction within the macrocycle. Both isomers **1a** and **1b** were present in the crude product also due to the irreversible nature of the macrocyclization reaction. This is in contrast with the common synthesis of bambusuril which employs reversible acidic conditions enabling a transformation of kinetic products to thermodynamic products [12].

Only a small quantity of macrocycle **2** in the crude mixture was identified. This minor product should be present in 4 isomeric forms, but we were not able to achieve separation of these isomers. Therefore, our attention turned to macrocycle **1**, which was the major product of the macrocyclization reaction. The separation of diastereomers **1a** and **1b** was achieved by preparative HPLC using a reversed-phase column and acetonitrile/water gradient elution. The pure isomers **1a** and **1b** were subjected to chemical-physical analysis.

**Figure 2:**
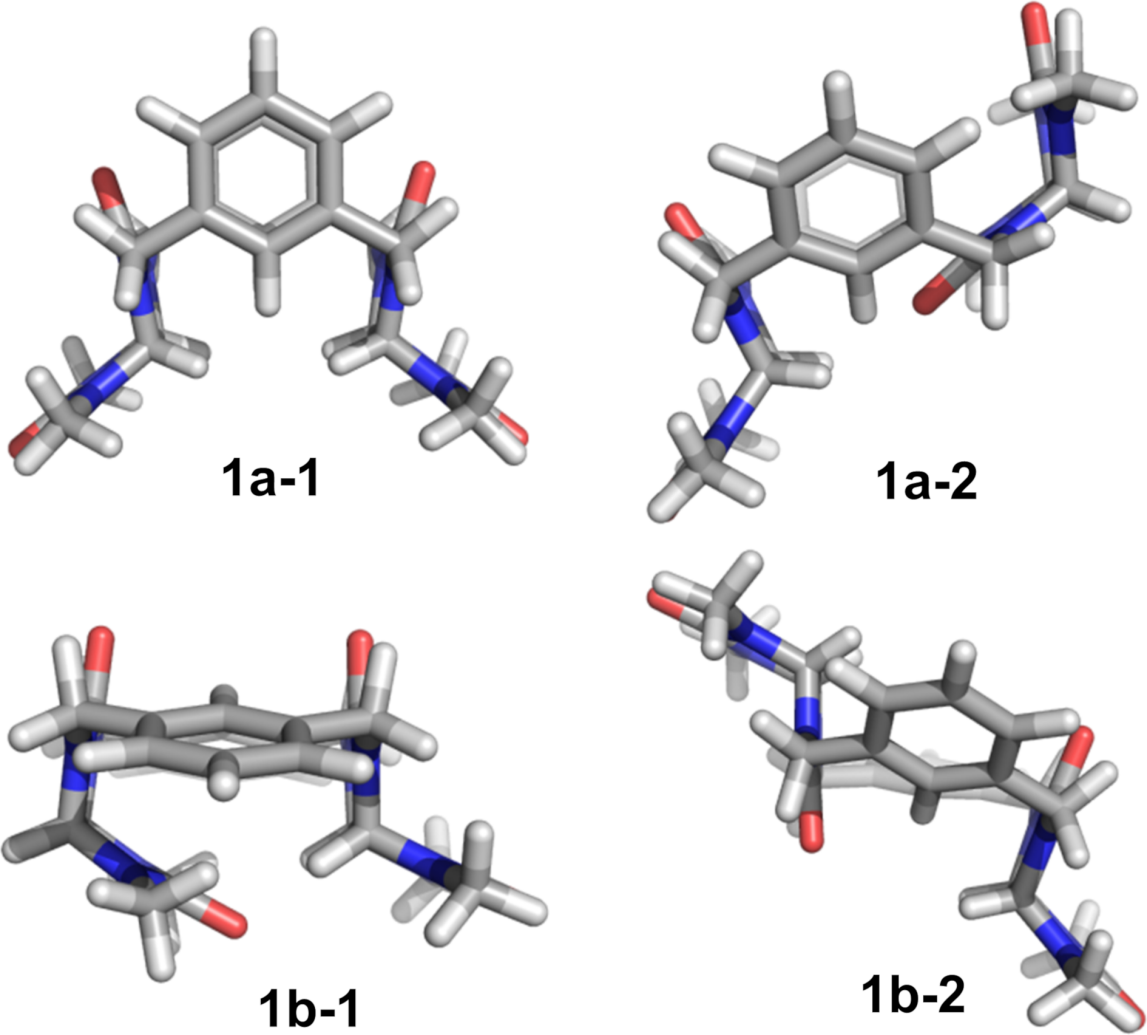
Conformers of macrocycles **1a**, **1b** optimized at the CAM-B3LYP/6-31G(d) level of theory.

We first measured ^1^H NMR spectra of **1a** in CD_3_CN at 30 °C (Supporting Information File 1, Figure S5), which showed broad signals of glycoluril and aromatic units. Since the glycoluril units have some degree of flexibility within the macrocycle, we envisioned that the signal broadening is caused by rapid interconversion between several preferred conformers of the macrocycle. The DFT optimization performed at the CAM-B3LYP/6-31G(d) level of theory revealed that **1a** can occur in form of two preferred conformers **1a-1** and **1a-2** (Figure 2).

**Figure 3:**
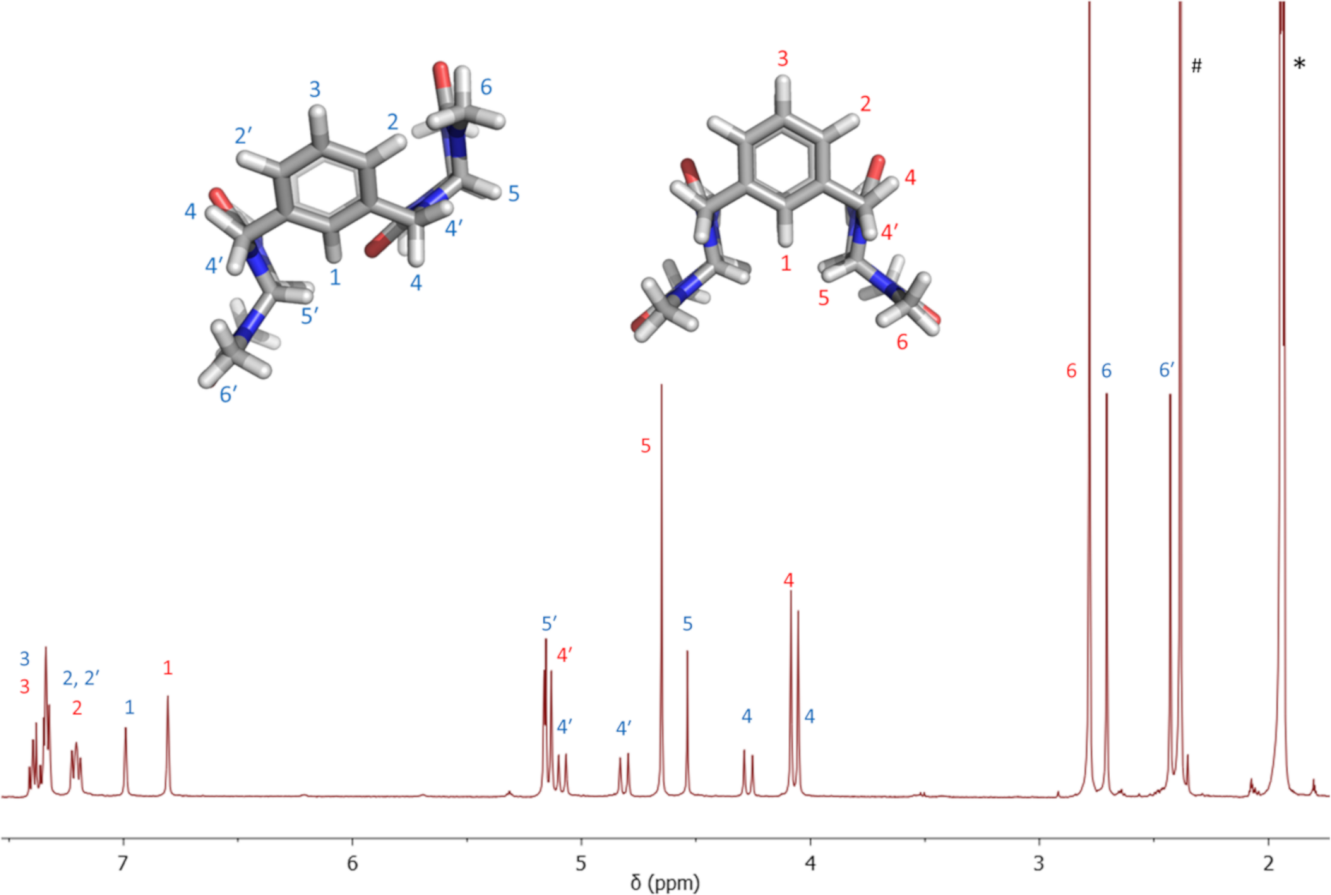
^1^H NMR spectrum of **1a** in MeCN-*d*_3_ measured at −40 °C. ^#^Signal of water. *Signal of acetonitrile.

The optimized structures provided us with support to understand the spatial arrangement of the conformers for further NMR studies. While broad signals of **1a** were obtained at 30 °C, lowering of the temperature resulted in sharpening of the signals in the ^1^H NMR spectra. The spectrum of **1a** measured at −40 °C in CD_3_CN showed two sets of signals (Figure 3) which were assigned to the calculated conformers. Conformer **1a-1** possesses a plane of symmetry, therefore, is characterized by single signals of methyl (H6), methine (H5) and aromatic (H1 and H3) protons. Contrary, **1a-2** is characterized by two signals for each of these protons because of its non-equivalent nature.

**Figure 4:**
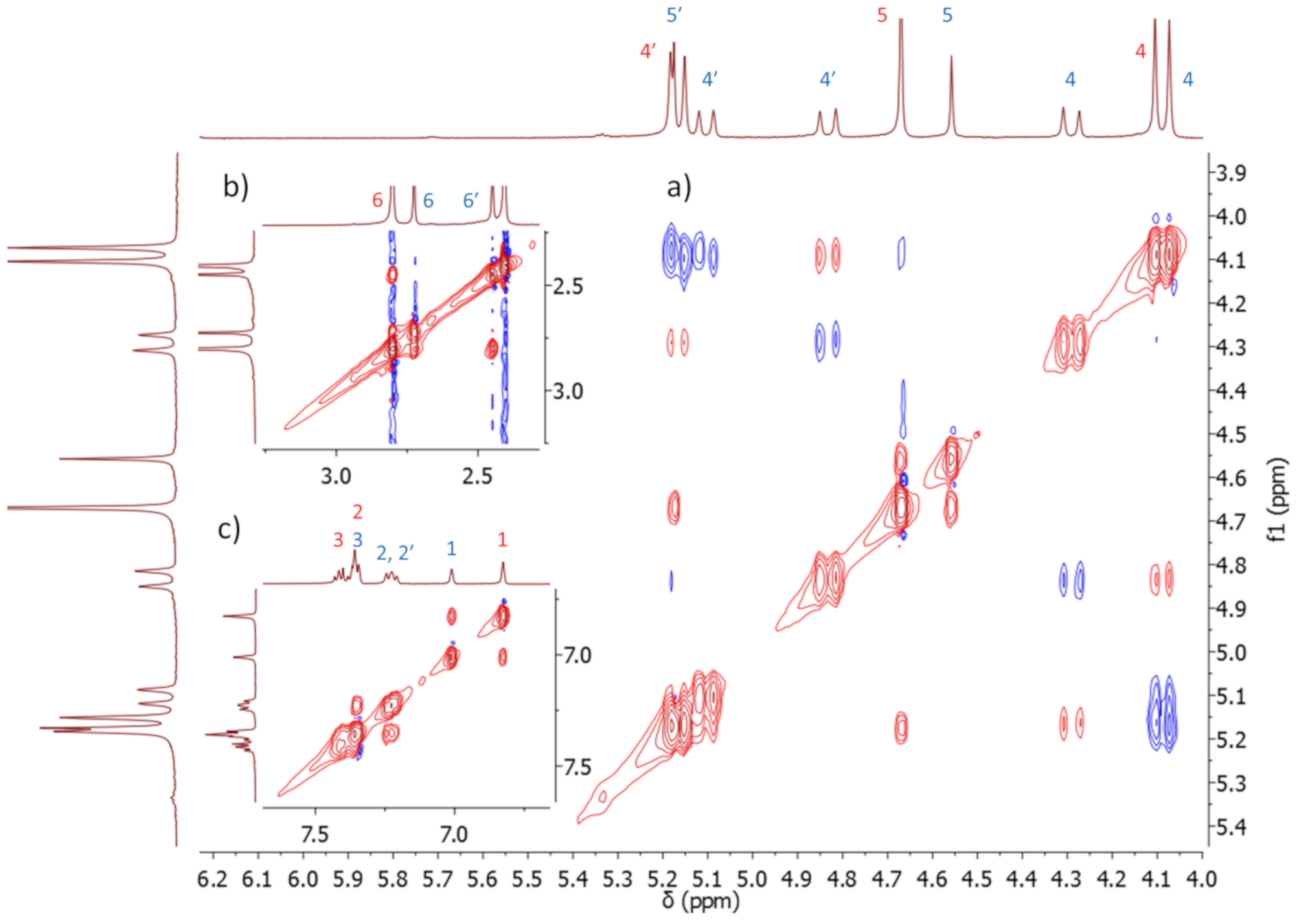
2D EXSY spectra (ROESY cross-peaks in blue, EXSY cross-peaks in red) measured in MeCN-*d*_3_ at −40 °C. a) Zoom of the methylene and methine region, b) zoom of methyl signals, c) zoom of aromatic signals.

The structures of conformers **1a-1** and **1a-2** were further confirmed by 2D NMR experiments. We performed 2D exchange spectrometry (EXSY) NMR experiments to confirm that there is a change of conformation on the NMR timescale [17]. This technique allows to correlate the exchanging signals in both slow and fast exchange regimes. The measured ROESY spectrum featured both NOE interactions of the opposite phase and EXSY peaks of the same phase as diagonal peaks. The EXSY cross-peaks indicate the chemical exchange between methylene protons (H4), the aromatic protons (H1) and methyl protons (H6, Figure 4). The NOE interaction between the glycoluril methine protons (H5) and aromatic proton (H1, Figure 4) also confirmed the assignment of **1a-1**. The integration of signals in ^1^H NMR spectra at −40 °C revealed uneven populations of conformers in a 4:3 ratio in favor of **1a-1** which remained constant at even lower temperature. By application of the Boltzmann distribution to the conformer population, we have calculated that **1a-1** is more stable by 0.13 kcal mol^−1^ than **1a-2**. Conformer **1a-1** is preferred probably due to its higher symmetry.

Diastereomer **1b** was characterized in the same manner as **1a**. The molecular modelling showed that **1b** also exists in two forms of conformers **1b-1**, **1b-2** differing by the position of the glycoluril units (Figure 2). The NMR spectra (Supporting Information File 1, Figure S9) measured at low temperature featured two sets of signals reflecting the presence of conformers **1b-2** and **1b-1**. However, the ratio of these conformers is 82:1 in favor of **1b-1** which translates to the energy difference of 2.04 kcal mol^−1^. Stabilization of the **1b-1** conformer can be attributed to the intramolecular hydrogen bonding interaction between the oxygen atom of one glycoluril unit and methine protons of the second glycoluril unit.

**Figure 5:**
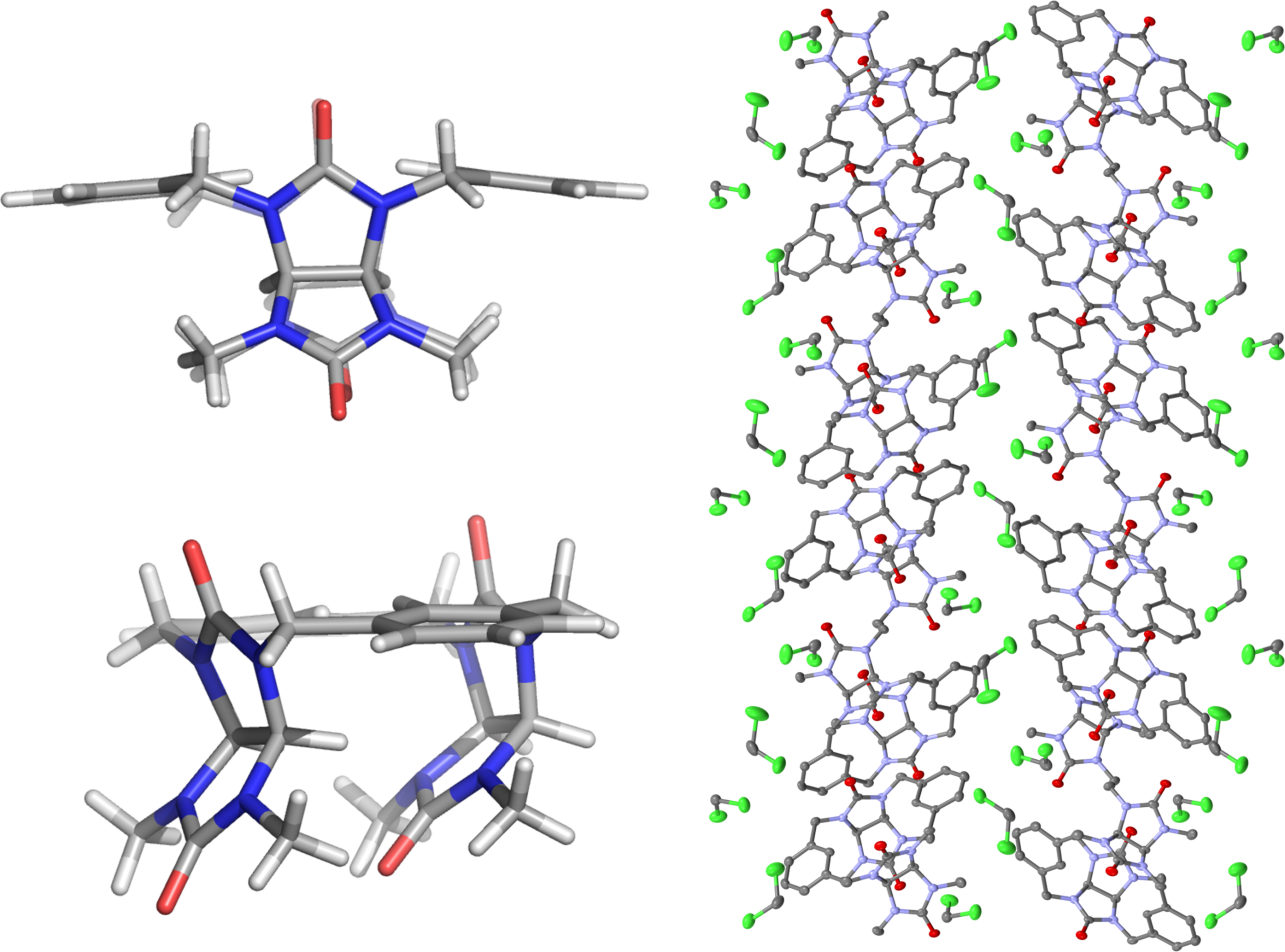
X-ray crystal structure of **1b-1** (left), layered structure of crystal packing (right).

The monocrystals of **1b** suitable for X-ray analysis were obtained upon slow diffusion of diisopropyl ether into the solution of **1b** in dichloromethane and benzene. The X-ray structure features *C*_s_ symmetry (Figure 5), which is in good agreement with the structure of the **1b-1** conformer calculated by DFT. The parallel glycoluril units are placed on one side of the plane defined by the xylylene moieties. The macrocycles form a layered structure stabilized by intermolecular hydrogen bonding interaction between the carbonyl oxygens of one molecule and methine and methylene hydrogen atoms of neighboring macrocycles.

Finally, we investigated the affinity of the newly prepared bambusuril analogs **1a** and **1b** towards anions. The addition of halide anions in form of their tetrabutylammonium salts to the solutions of **1a** and **1b** did not induced any change in the NMR spectra of the macrocycles. This indicates that these macrocycles do not bind the studied anions. We assume that the cavity of the macrocycles is too small to be able to include any anions. Indeed the conformer **1b-1** with two facing glycolurils resembles the bambus[4]uril, which also cannot bind anions due to its small cavity [18].

## Conclusion

The reaction between 2,4-dimethylglycoluril and *m*-xylylene dibromide under basic conditions yielded a mixture of macrocycles, from which the major product **1** was isolated. Two diastereomers of the macrocycle, **1a** and **1b**, differing in the orientation of one glycoluril unit were obtained. The building blocks within the macrocycles connected via methylene bridges are flexible giving rise to two preferred conformers for each diastereomer. These conformers were identified by molecular modelling and also by low-temperature NMR experiments. Unfortunately, both macrocycles **1a** and **1b** failed to act as an anion receptors due to their small cavities. Therefore, our future experiments will focus on further optimization of the reaction conditions and on the isolation of the three-membered macrocycle suitable for anion binding.

## Experimental

### General remarks

The commercially available starting materials and reagents were used without further purification. 2,4-Dimethylglycoluril was prepared according to a published procedure [11]. ^1^H and ^13^C NMR spectra were recorded with a Bruker Avance 500 spectrometer with working frequencies of 500.13 MHz (^1^H) and 125.77 MHz (^13^C). The spectrometer was equipped with a BBFO probe. Two-dimensional exchange spectroscopy (EXSY) spectra were recorded with a mixing time of 0.1 s. The experiments were recorded at 233.15 K unless stated otherwise. Chemical shifts are given in parts per million (ppm) and referenced to solvent residual peaks [19], the coupling constants (*J*) are reported in hertz (Hz). The multiplicities of signals are reported as singlet (s), doublet (d), triplet (t), multiplet (m). The flash chromatography was performed on a Büchi Reveleris PREP Purification System using RP C18 40g cartridges. Preparative HPLC was done by an Agilent 1260 Infinity using a VP Nucleodur 250/21 C18 HTEC 5 µm column. The gradient separation was achieved for both using HPLC grade MeCN and milli-Q water. High-resolution mass spectra were recorded by an accurate-mass TOF LC/mass spectrometer using multimode ESI/APCI as an ion source and a manual pump for sampling. Matrix-assisted laser desorption ionization–time-of-flight mass spectra (MALDI–TOF MS) were measured on a MALDI–TOF MS UltrafleXtreme (Bruker Daltonics) and samples were ionized with the aid of a Nd:YAG laser (355 nm) from α-cyano-4-hydroxycinnamic acid (HCCA) matrix. Diffraction data were collected at 120 K on a diffractometer with graphite-monochromated Mo Kα radiation.

### Macrocycles **1a** and **1b**

2,4-Dimethylglycoluril (1.70 g, 10 mmol) and LiClO_4_ (1.06 g, 10 mmol) were suspended in dry THF (200 mL) under an Ar atmosphere. After addition of LiH (0.47 g, 60 mmol) the mixture was heated to reflux for 4 hours then cooled to 0 °C. *m*-Xylylene dibromide (2.65 g, 10 mmol) was added in three portions. The mixture was stirred at 0 °C for 2 hours then heated to reflux for 6 days. The reaction was quenched by the addition of 40 mL of water. THF was evaporated under reduced pressure and precipitated solid material was filtered off. The crude product was dried under vacuum.

The crude product was purified by GRACE flash chromatography using a C-18 reversed-phase cartridge and acetonitrile/water as mobile phase. The sample (350 mg of crude product) was dissolved in DMSO (400 μL) and applied to the column. At the flow rate of 20 mL/min the gradient elution went from 20% MeCN to 45% MeCN in 30 min, followed by gradient to 100% MeCN in 5 min, then continued with MeCN. The macrocycle **1** eluted at *t* = 12.5 min in 49 mg yield (14%).

The diastereomers of **1** were further separated using preparative HPLC. The sample (30 mg of **1**) was dissolved in DMSO (500 μL) and applied to the column. At the flow rate 40 mL/min the gradient elution went from 10% MeCN to 70% MeCN in 35 min, followed by gradient to 100% MeCN in 5 min, then continued with MeCN. The macrocycle **1a** eluted at *t* = 27.2 min, macrocycle **1b** eluted at *t* = 28.1 min.

**1a: **^1^H NMR (500 MHz, acetonitrile-*d*_3_) δ 7.43–7.30 (m, 6H), 7.25–7.17 (m, 4H), 6.99 (s, 2H), 6.81 (s, 2H), 5.16 (s, 2H), 5.15 (d, *J* = 15.7 Hz, 4H), 5.08 (d, *J* = 16.0 Hz, 2H), 4.81 (d, *J* = 17.7 Hz, 2H), 4.65 (s, 4H), 4.54 (s, 2H), 4.27 (d, *J* = 17.7 Hz, 2H), 4.07 (d, *J* = 15.8 Hz, 4H + 2H), 2.78 (s, 12H), 2.71 (s, 3H), 2.43 (s, 6H); ^13^C NMR (126 MHz, acetonitrile-*d*_3_) δ 140.25, 137.91, 137.05, 130.00, 129.39, 128.96, 127.30, 125.98, 124.81, 122.59, 73.31, 68.47, 68.08, 46.95, 45.32, 45.18, 31.19, 31.00, 29.75; HRMS (APCI^+^) *m*/*z*: [M + H]^+^ calcd for C_28_H_32_N_8_O_4_, 545.2619; found, 545.2616.

**1b: **^1^H NMR (500 MHz, acetonitrile-*d*_3_) δ 7.37 (t, *J* = 7.5 Hz, 2H), 7.24 (t, *J* = 7.5 Hz, 4H), 6.87 (s, 2H), 5.15 (d, *J* = 16.6 Hz, 2H), 5.10 (d, *J* = 16.9 Hz, 4H), 4.58 (s, 2H), 4.15 (d, *J* = 16.7 Hz, 2H), 4.10 (d, *J* = 16.8 Hz, 2H), 2.65 (s, 6H), 2.00 (s, 6H); ^13^C NMR (126 MHz, acetonitrile-*d*_3_) δ 140.84, 136.56, 129.65, 127.21, 126.72, 123.21, 73.32, 68.34, 45.94, 44.83, 31.15, 30.39; HRMS (APCI^+^) *m*/*z*: [M + H]^+^ calcd for C_28_H_32_N_8_O_4_, 545.2619; found, 545.2618.

## Supporting Information

File 1 MS and NMR spectra, computational details and crystallographic data for macrocycles **1a** and **1b**.

File 2 CIF data for compound **1b-1**.
