# Supplementary material for: Bambusuril analogs based on alternating glycoluril and xylylene units
Source: Beilstein J Org Chem. 2019 Jun 11;15:1268–74. doi: 10.3762/bjoc.15.124 (PMC6604679; doi:10.3762/bjoc.15.124)
Supplement: File 1 — MS and NMR spectra, computational details and crystallographic data for macrocycles 1a and 1b. [file Beilstein_J_Org_Chem-15-1268-s001.pdf]

BEILSTEIN JOURNAL OF ORGANIC CHEMISTRY

Supporting Information

for

Bambusuril analogs based on alternating glycoluril and xylylene units

Tomáš Lízal and Vladimír Šindelář

Beilstein J. Org. Chem. **2019,** *15,* 1268–1274. doi:10.3762/bjoc.15.124

MS and NMR spectra, computational details and crystallographic data for macrocycles 1a and 1b

License and Terms: This is a supporting information file under the terms of the Creative Commons Attribution License (http://creativecommons.org/licenses/by/4.0). Please note that the reuse, redistribution and reproduction in particular requires that the authors and source are credited.
The license is subject to the *Beilstein Journal of Organic Chemistry* terms and conditions: (https://www.beilstein-journals.org/bjoc)

Table of contents

MALDI: S2

HPLC S2

HR-MS: S3

NMR S4

Determination of energy difference of conformers: S7

Computational details S8

X-ray crystallography S8

MALDI:

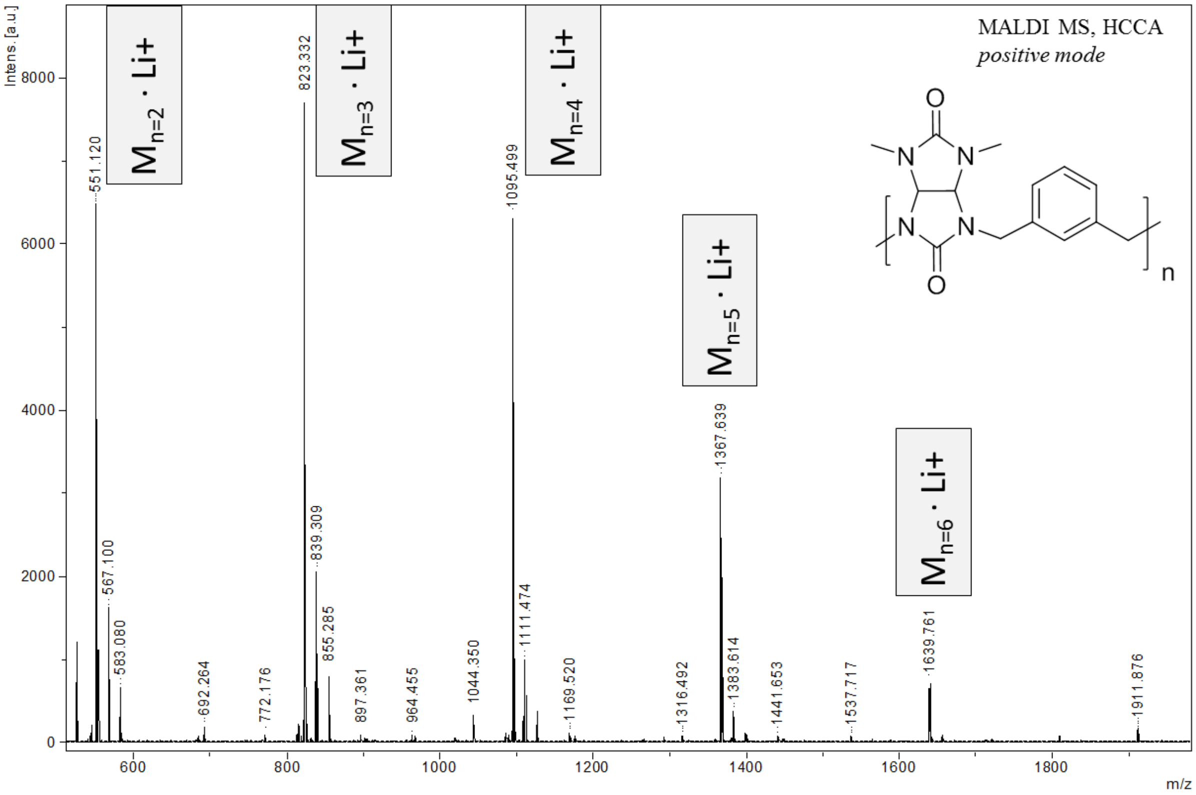

Figure S1: MALDI MS spectra of the crude product.

HPLC

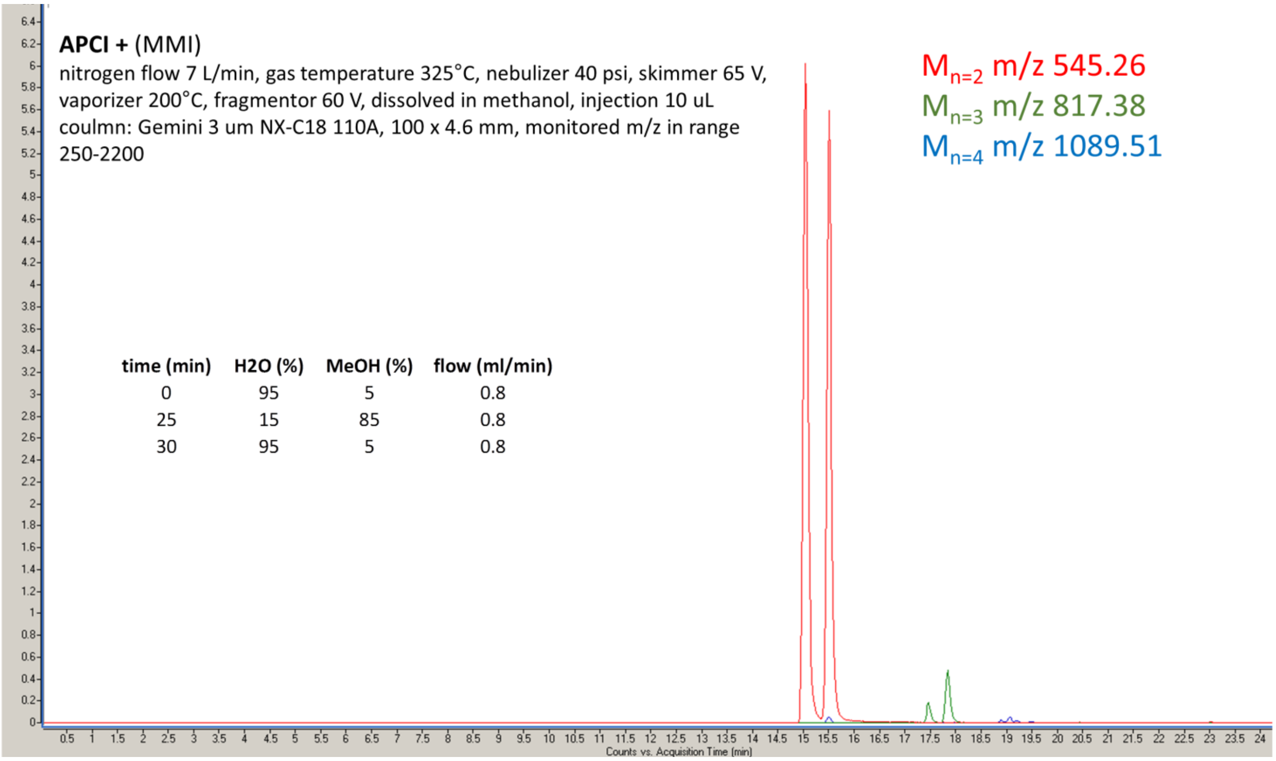

Figure S2: HPLC-MS analysis of the crude product.

HR-MS:

$C_{28}H_{32}N_8O_4$ **exact mass: 544.2547**

APCI + (MMI)
nitrogen flow 5 L/min, gas temperature 325°C, nebulizer 45 psig, skimmer 65 V,
vaporizer 200°C, fragmentor 40 V, dissolved in methanol

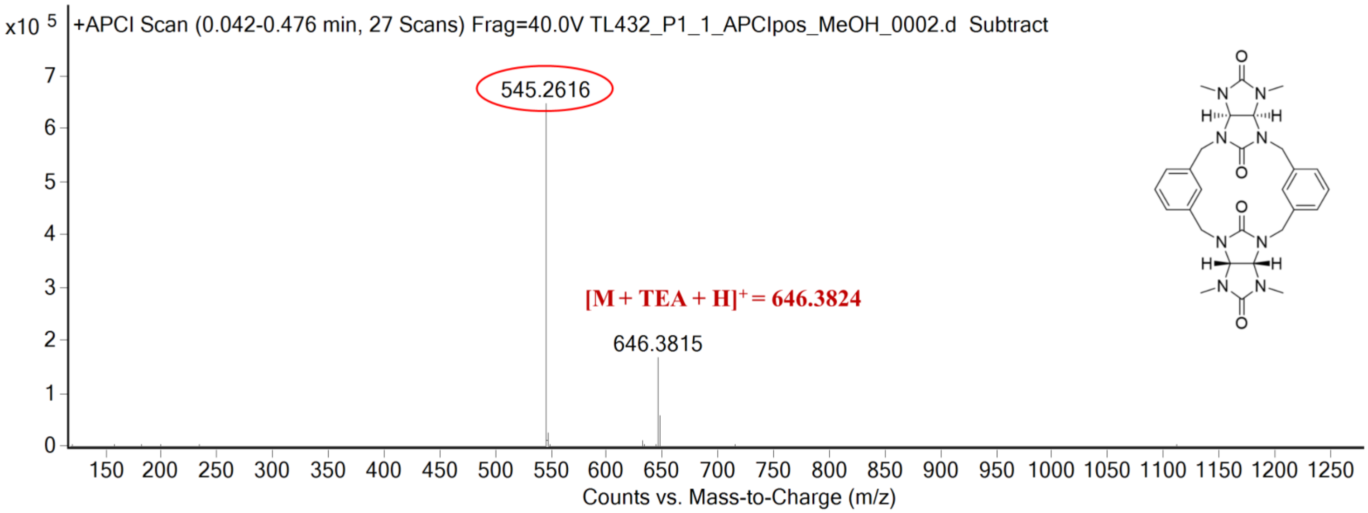

expected mass: $[M+H]^+$ = 545.2619   observed mass: $[M+H]^+$ = 545.2616   mass accuracy = - 0.6 ppm

Figure S3: HR-MS analysis of **1a**.

$C_{28}H_{32}N_8O_4$ **exact mass: 544.2547**

APCI + (MMI)
nitrogen flow 5 L/min, gas temperature 325°C, nebulizer 45 psig, skimmer 65 V,
vaporizer 200°C, fragmentor 20 V, dissolved in methanol

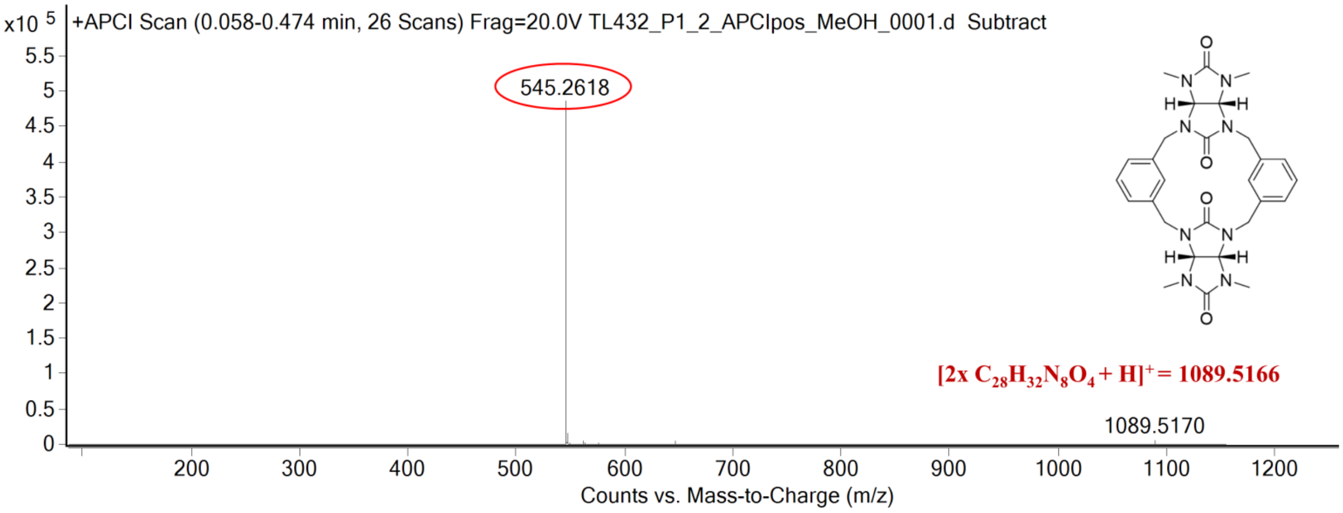

expected mass: $[M+H]^+$ = 545.2619   observed mass: $[M+H]^+$ = 545.2618   mass accuracy = - 0.2 ppm

Figure S4: HR-MS analysis of **1b**.

NMR:

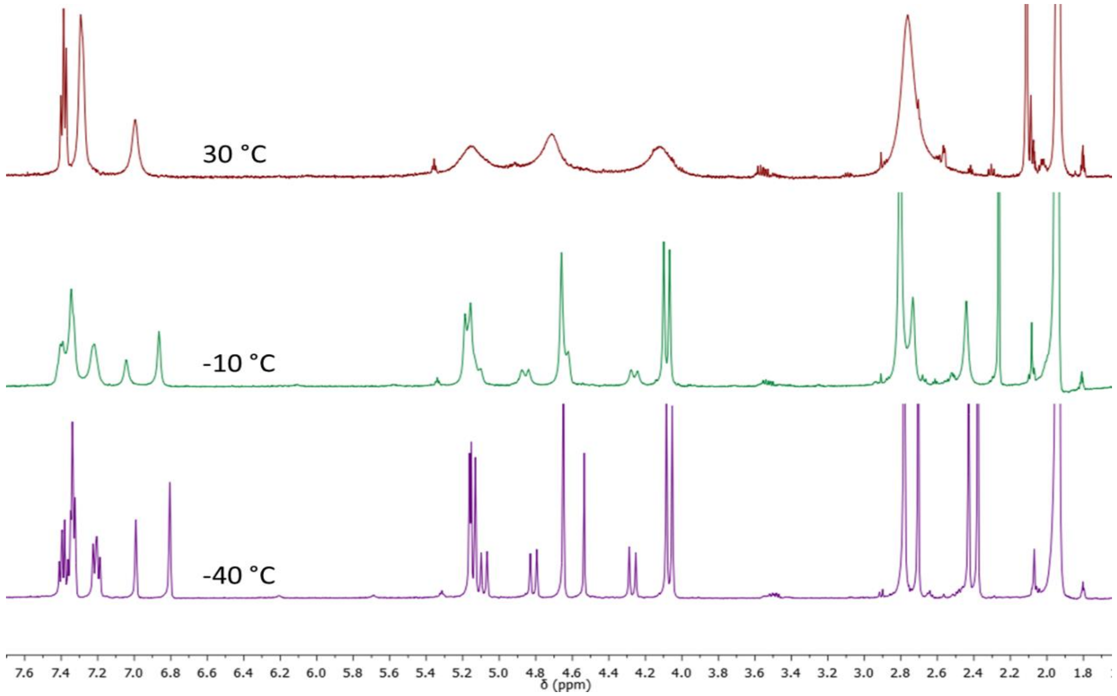

Figure S5: Variable-temperature ^{1}H NMR spectrum of **1a** measured at 30 °C, −10 °C and −40 °C in MeCN-d_3.

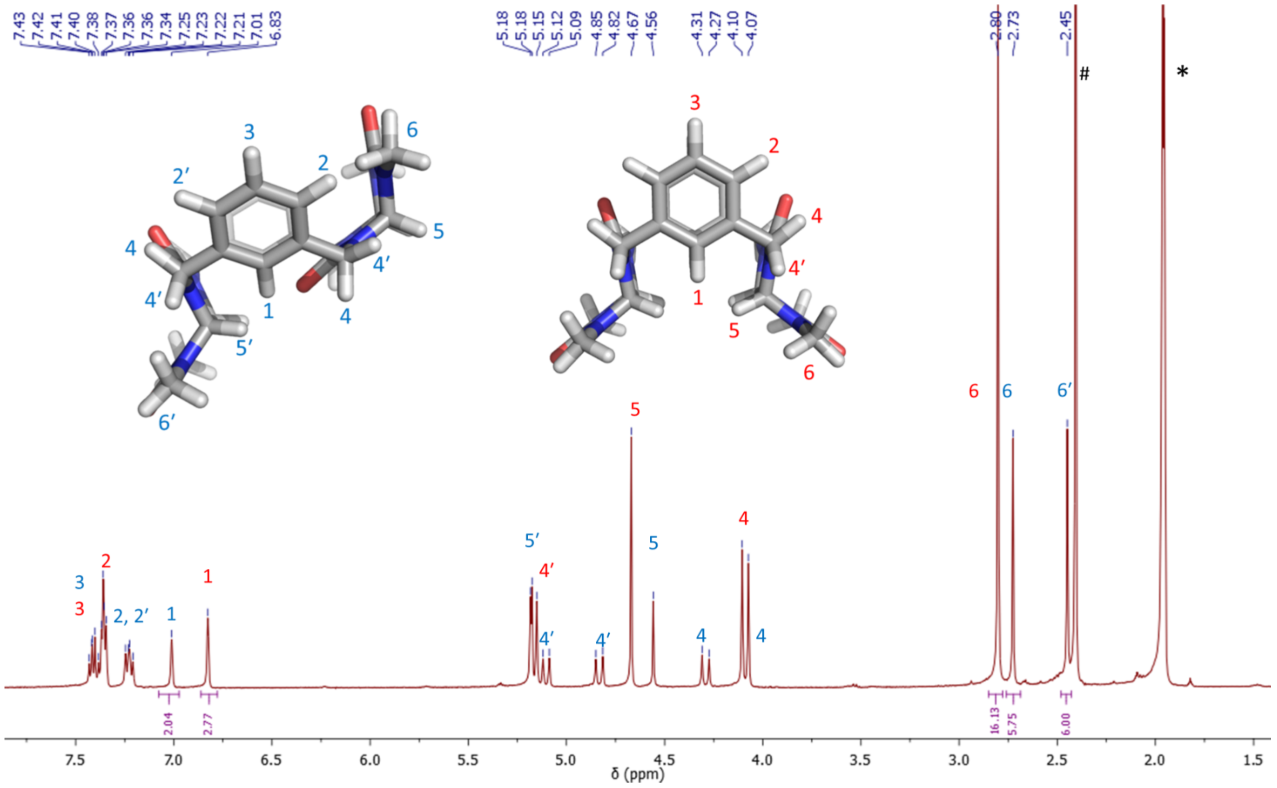

Figure S6: ^{1}H NMR spectrum of **1a** measured at −40 °C in MeCN-d_3 , #HDO signal, *MeCN signal.

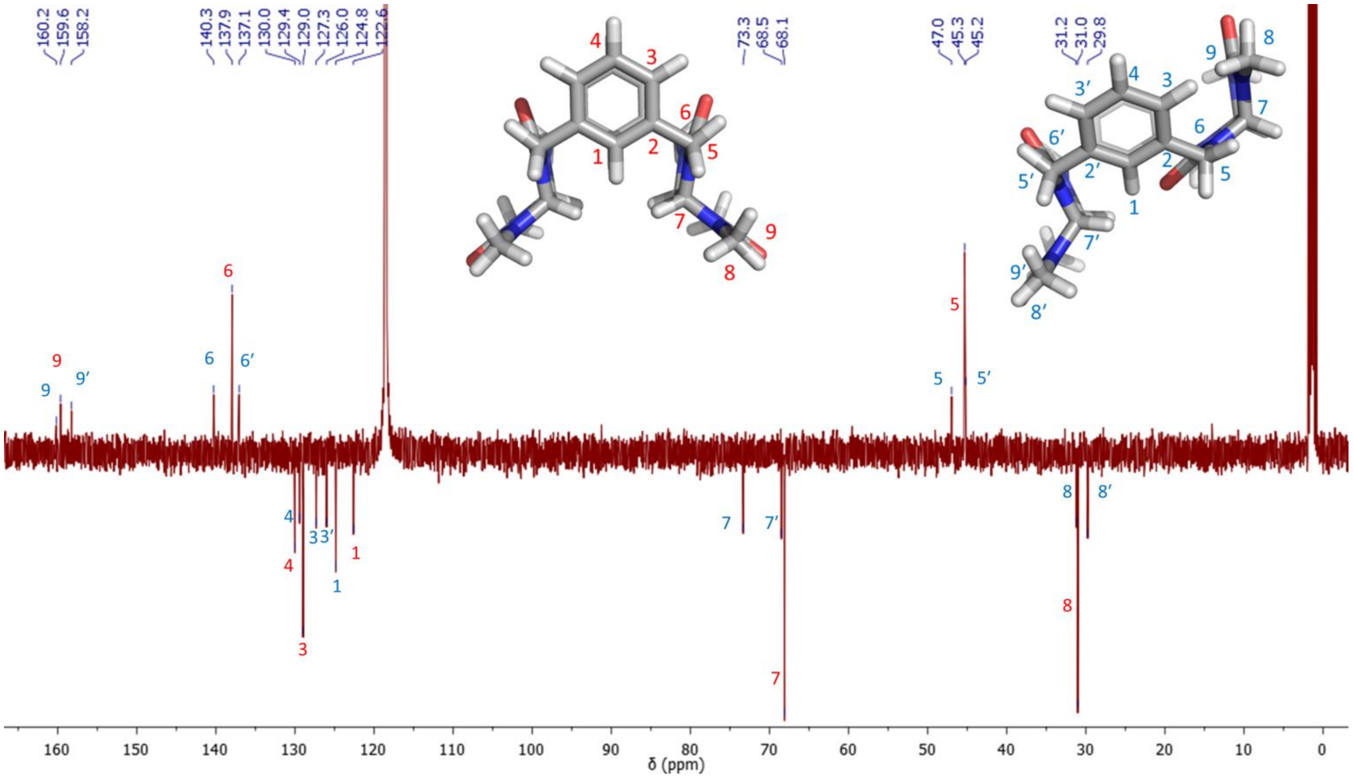

Figure S7: ^{13}C APT NMR spectrum of **1a** measured at −40 °C in MeCN-d_3.

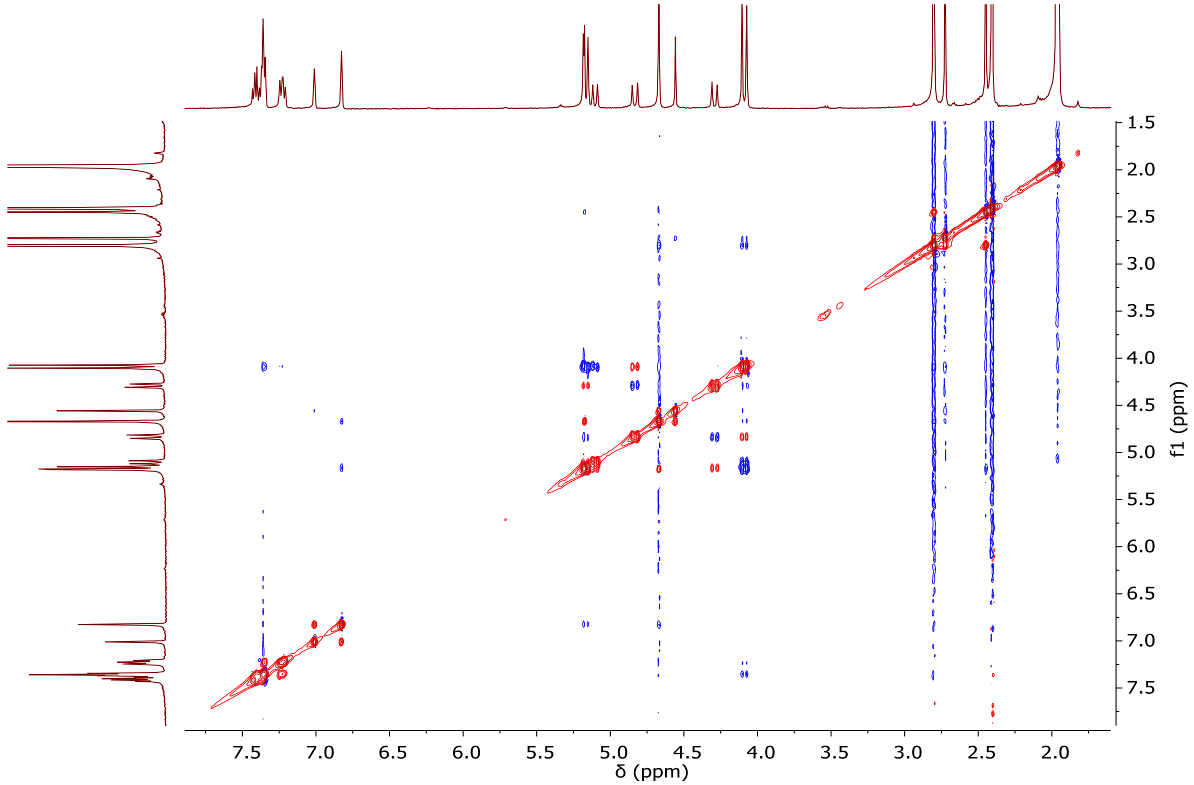

Figure S8: ROESY NMR spectrum of **1a** measured at −40 °C in MeCN-d_3.

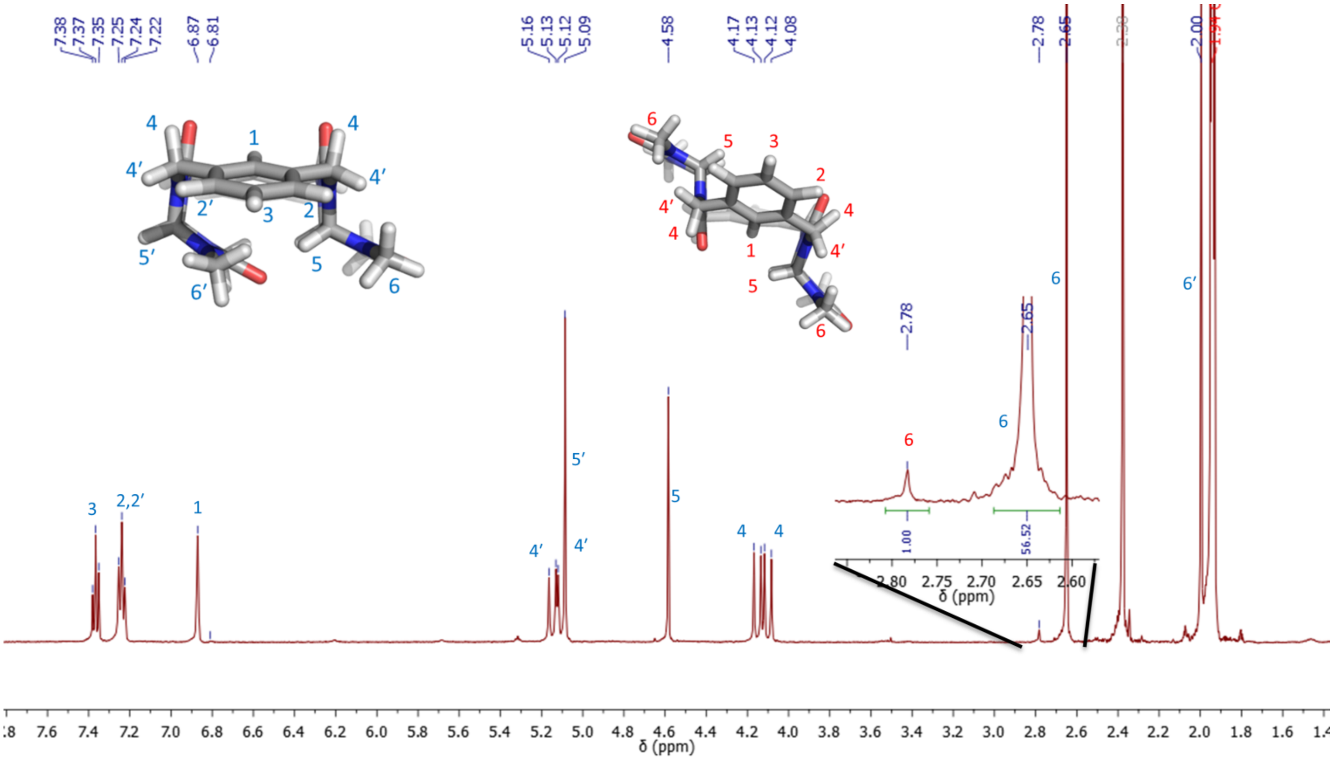

Figure S9: ^{1}H NMR spectrum of **1b** measured at −40 °C in MeCN-d_3.

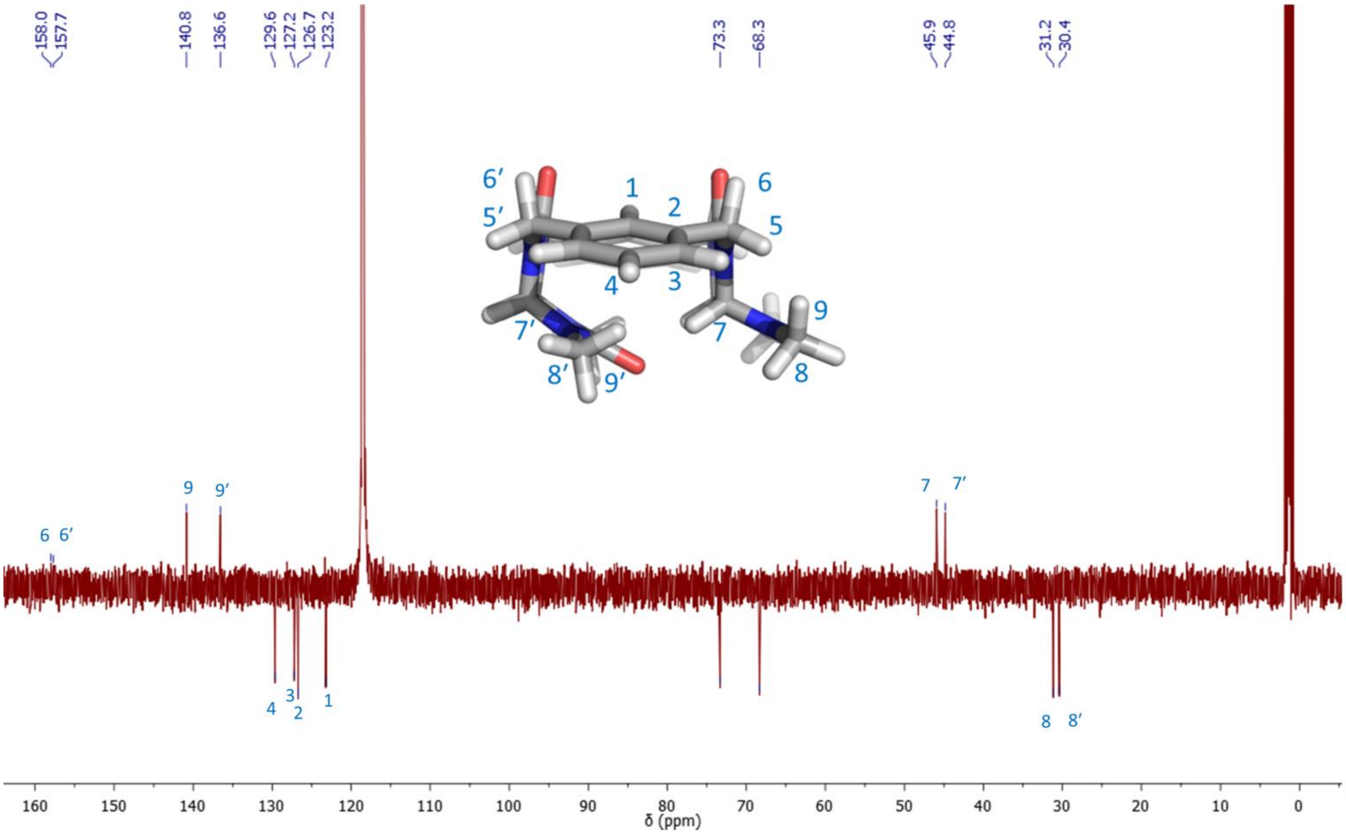

Figure S10: ^{13}C APT NMR spectrum of **1b** measured at −40 °C in MeCN-d_3.

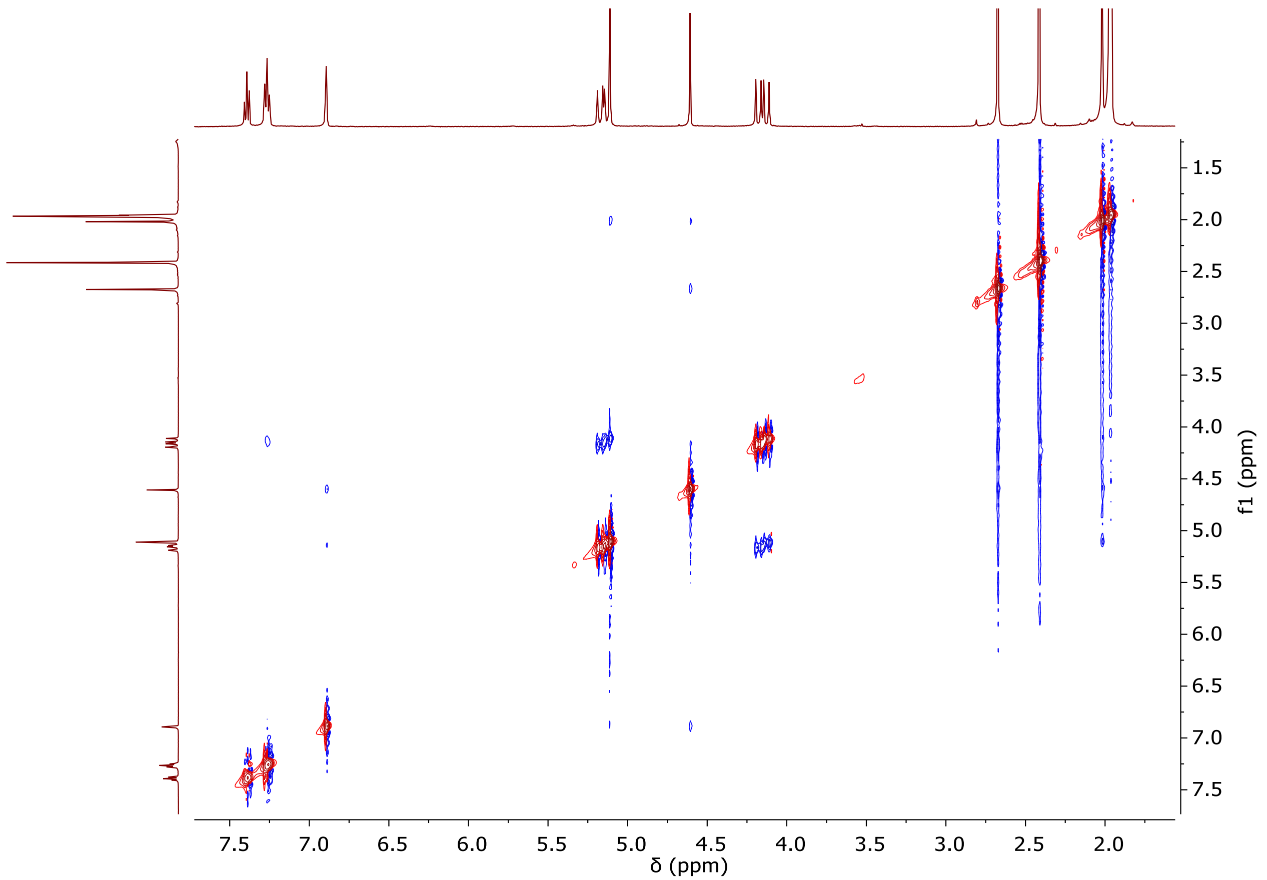

Figure S11: ROESY NMR spectrum of **1b** measured at −40 °C in MeCN-d_3.

Determination of energy difference of conformers:

Application of Boltzmann equation:

The energy differences in of the conformers were calculated using equation (1)[1],

$$\frac{N_i}{N_j} = e^{\frac{-(E_i - E_j)}{kT}} \rightarrow \ln\frac{N_i}{N_j} kT = -(E_i - E_j) \quad (1)$$

where N_i is the population of conformer i, N_j is the population of conformer j, k is the Boltzmann constant (kcal mol^{-1}), T is the temperature of measurement (233.15 K).

1a (MeCN, -40 °C): $\ln\frac{4}{3} 0.001987 \times 233.15 = -(E_i - E_j) = 0.13\, kcal/mol$

1b (MeCN, -40 °C): $\ln\frac{82}{1} 0.001987 \times 233.15 = -(E_i - E_j) = 2.04\, kcal/mol$

Computational details

Computational methods available in Spartan ’18 software[2] were employed to determine geometries and properties of macrocycle conformers. The structures of **1a-1**, **1a-2, 1b-1 and 1b-2** were built in silico. The geometry of the structures were optimized at the CAM-B3LYP/6-31G(d) level of theory using C-PCM solvation model of acetonitrile.

X-ray crystallography

Diffraction data were collected at 120 K on Rigaku Saturn944+ diffractometer with graphite-monochromated Mo Kα radiation. The structures were solved by direct methods and refined using ShelXTL software package[3].

CCDC No.	1898386
Empirical formula	$C_{30}H_{36}Cl_4N_8O_4$
Formula weight	714.47
Crystal system	Orthorhombic
Space group	$Pca2_1$
a [Å]	30.482(10)
b [Å]	9.034(3)
c [Å]	22.961(7)
Volume [$Å^3$]	6323(4)
Z	8
μ [mm^{-1}]	0.426
Crystal size [mm]	0.18 × 0.13 × 0.10
θ range [°]	1.1–27.5
Reflections collected/unique	19956/ 9563
R_{int}	0.042
Data/restraints/parameters	9563/745/837
Final *R* indices [$I>2\sigma(I)$]	0.045
$\Delta\rho_{max}$ /$\Delta\rho_{min}$ [e $Å^{-3}$]	0.47/ −0.47

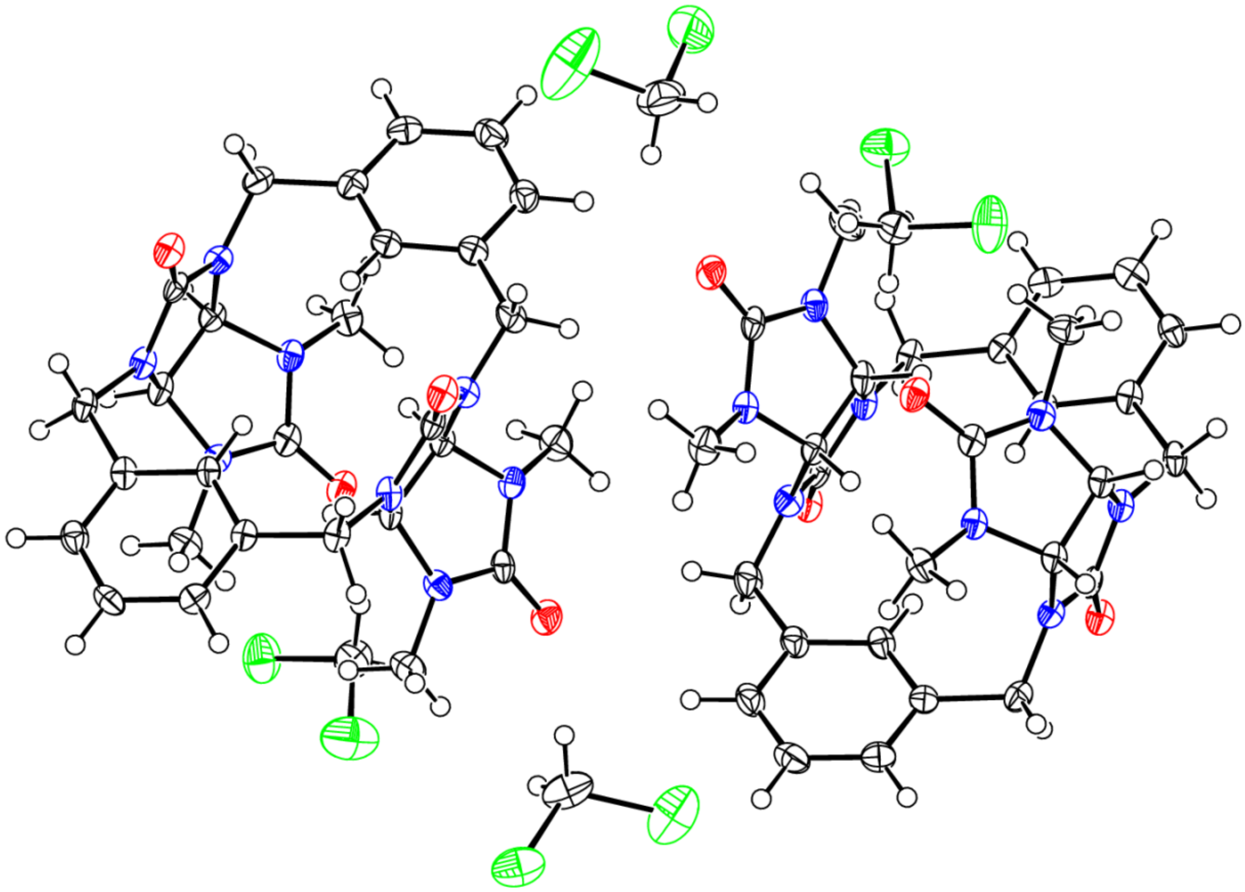

Figure S12: Molecular structure of **1b-1**. Thermal ellipsoids are drawn at the 50% probability level.

References:

(1) Anslyn, E. V.; Dougherty, D. A. *Modern Physical Organic Chemistry*; University Science, 2006.
(2) *Spartan'18*; Wavefunction: Irvine, CA, USA, 2018.
(3) Sheldrick, G. M. Crystal Structure Refinement with *SHELXL*. *Acta Crystallogr. Sect. C Struct. Chem.* **2015**, *71* (1), 3–8. https://doi.org/10.1107/S2053229614024218.
